# Annual evolution of the prescription of drugs with prognostic implications in acute decompensated heart failure with reduced ejection fraction

**DOI:** 10.1186/s12872-024-03728-y

**Published:** 2024-02-14

**Authors:** Raquel López-Vilella, Víctor DonosoTrenado, Borja Guerrero Cervera, Ignacio Sánchez-Lázaro, Luis Martínez Dolz, Luis Almenar Bonet

**Affiliations:** 1 Heart Failure and Transplant Unit, Hospital Universitari i Politècnic La Fe, Valencia, 46026 Spain; 2 Cardiology Department, Hospital Universitari i Politècnic La Fe, Valencia, 46026 Spain; 3 Centro de Investigación Biomédica en Red de Enfermedades Cardiovasculares (CIBERCV), Instituto de Salud Carlos III, Madrid, Spain

**Keywords:** Quadruple therapy, Acute heart failure, Heart failure subgroups, Prescription, Evolution

## Abstract

**Background:**

Quadruple therapy (renin angiotensin system inhibitors, beta-blockers, mineralocorticoid receptor antagonists and sodium/glucose cotransporter type 2 inhibitors [SGLT2i]) has become the current prognostic modifying treatment for heart failure (HF) with reduced ejection fraction (HFrEF). This study aimed to analyse the prescription´s evolution of this combination therapy, the analysis of each pharmacological group and the differences according to HF subgroups.

**Methods:**

Retrospective analysis of consecutive patients admitted for cardiac decompensation. Inclusion period: from 1-1-2020 to 12-31-2022. Patients with left ventricular ejection fraction > 40% and deceased during admission were excluded. Finally, 602 patients were included. These were divided into: (a) de novo HF without previous heart disease (n:108), (b) de novo with previous heart disease (n:107), and (c) non-de novo (n:387).

**Results:**

Over the study time, all pharmacological groups experienced an increase in drugs prescription (*p* < 0.001). The group with the largest prescription rate increase was SGLT2i (2020:20%, 2021:42.9%, 2022:70.4%; mean increase 47.2%). The discharge rate prescription of quadruple therapy increased progressively (2020:7.4%, 2021:21.1%, 2022:32.5%; mean increase 21.9%). The subgroup with the highest combined prescription in 2022 was de novo with previous heart disease (43.9%).

**Conclusion:**

The pharmacological group with the largest prescription´s rate increase was SGLT2i. The percentage of patients discharged on quadruple therapy has progressed significantly in recent years, although it remains low. The most optimised subgroup at discharge was that of de novo HF with previous heart disease.

## Background

Since the publication of clinical practice guidelines for the treatment of heart failure (HF) with reduced ejection fraction (HFrEF), quadruple therapy has become the optimal treatment that improves prognosis, reduces hospitalisations, and improves patients’ quality of life [1–3]. These 4 pharmacological groups are angiotensin-converting enzyme inhibitors/angiotensin receptor blockers/neprilysin inhibitors (ACEI/ARB/ARNI), beta-blockers (Bb), mineralocorticoid receptor antagonists (MRAs) and sodium/glucose cotransporter type 2 inhibitors (SGLT2i). All of them are included in the guidelines with the highest class of recommendation and level of evidence [1, 2]. Therefore, there is now agreement and a recommendation to incorporate these 4 pillars into the treatment of HF patients as soon as possible. However, the guidelines do not clearly state how to initiate treatment or how many pillars can actually be incorporated during the acute phase of decompensation. This is especially confusing with the last pharmacological group (SGLT2i) whose incorporation has been recent as the first clinical trial demonstrating its prognostic benefit in HF was published in 2019 [4].

The percentage of patients discharged from the hospital after an acute decompensation with the 4 drugs varies widely in the literature [5, 6]. This is probably due not only to methodological variations in the different studies but also because not all patients admitted with decompensation are the same and can be classified into at least 3 groups: de novo HF without previous heart disease, de novo with previous heart disease, and non-de novo.

This study hypothesized that the percentage of patients discharged after admission for decompensation with the 4 pillars would have increased in recent years, but could be insufficient. This percentage would be different according to the HF group (de novo without previous heart disease, de novo with previous heart disease, and non-de novo).

The primary objective of the study was to determine, in a large consecutive series of patients admitted for acute HF with reduced ejection fraction, the degree of implementation of treatment with the 4 pillars from admission to discharge. The secondary objectives were to compare the increase per year, pharmacological group, and study group.

## Methods

Retrospective analysis of a database of patients consecutively admitted to the Cardiology Department of a referral hospital with a diagnosis of HF. The database was filled in on the day the patient was discharged from hospital. To minimise errors, data collection, and database entry was performed by staff with expertise in the management of these patients and always by the same cardiologists from the HF Unit.

The inclusion period was from 1-1-2020 to 12-31-2022. This period was chosen because it was in 2019 when the implementation of SGLT2i as the fourth pillar of HF treatment was initiated following the DAPA-HF trial [4].

The 2021 European Society of Cardiology HF guidelines were followed for the diagnosis of acute HF [1]. Acute HF refers to rapid or gradual onset of symptoms and/or signs of heart failure, severe enough for the patient to seek urgent medical attention, leading to an unplanned hospital admission or an emergency department visit.

**Fig. 1:**
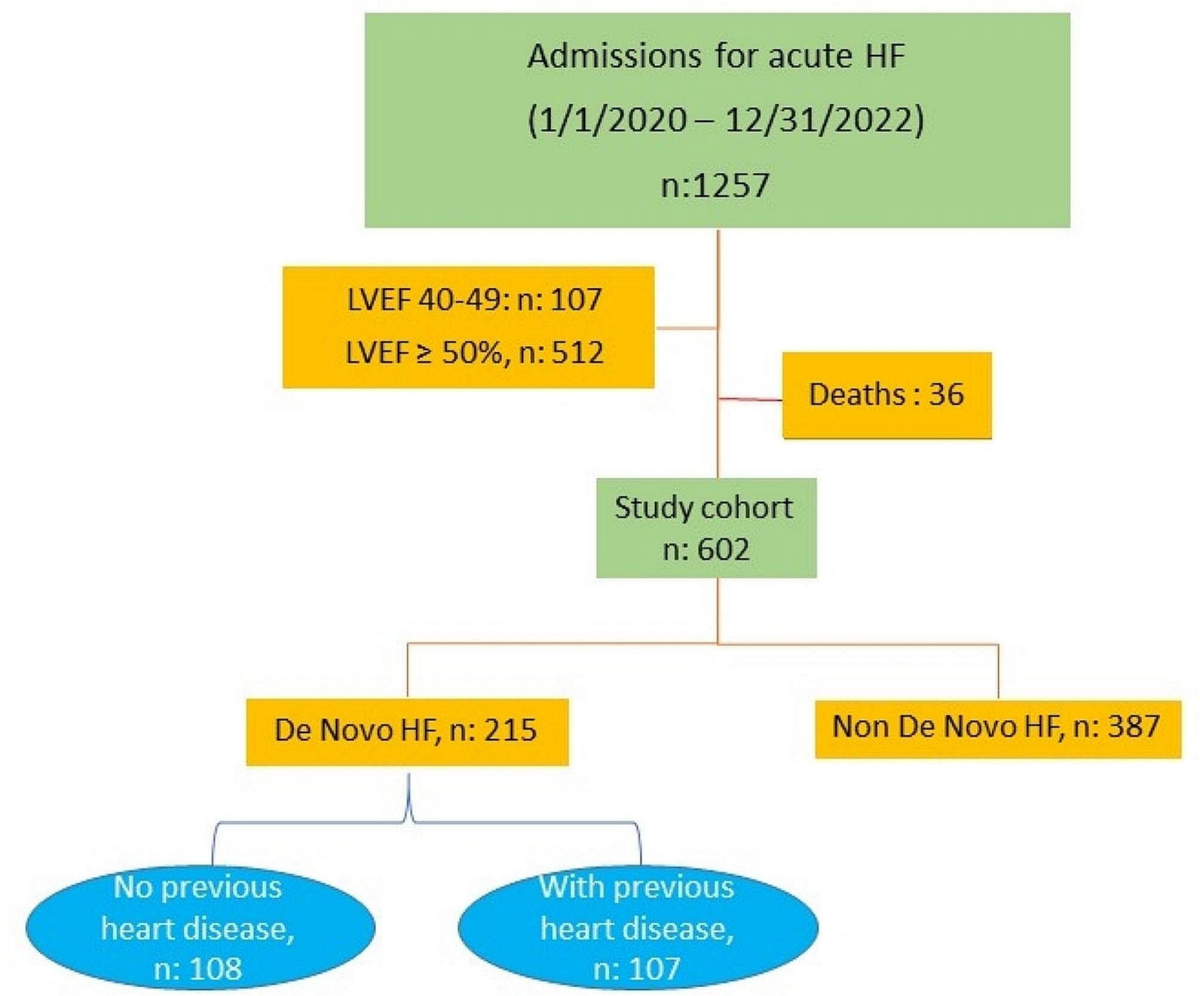
Flow chart Abbreviations: HF: heart failure; LVEF: left ventricular ejection fraction

Patients with left ventricular ejection fraction (LVEF) of 40–49% (n: 107), with LVEF ≥ 50% (n: 512), and patients who died during admission (n: 36) were excluded. The total number of patients analysed was 602. Patients were divided into three groups: (a) without a previous diagnosis of HF and without previous heart disease (n: 108), (b) without a previous diagnosis of HF but with any kind of previous heart disease (n: 107), (c) with a previous diagnosis of HF before hospital admission (n: 387). Patients with de novo HF but with previous heart disease were considered to be those with diagnosed heart disease (e.g. pre-existing ischaemic heart disease) but who had not presented HF until that time, with the selected admission being the debut of HF. The selection and distribution of patients can be seen in Fig. 1.

The key variables analysed were those related to treatment at admission and discharge to compare the percentage of prescription. The drugs analysed were renin-angiotensin-aldosterone system inhibitors [RAASi], including ACEIs, ARBs, and ARNI (MRA are analysed separately), MRAs, Bb, and SGLT2i. Clinical, echocardiographic, and analytical variables analysed overall and by study groups were collected and compared.

The study was approved by the hospital’s Biomedical Research Ethics Committee and followed the ethical principles for medical research on human subjects as defined by the Declaration of Helsinki. The Ethics Committee granted the study exemption from informed consent for non-deceased patients, due to the observational, non-interventional and retrospective nature of the study.

### Statistical analysis

To describe the sample characteristics, we used the absolute (n) and relative frequency (%) for qualitative variables and mean (standard deviation) for the quantitative variables if normal (Kolmogorov-Smirnov test) or median (25th and 75th percentiles) otherwise. Then, comparison among the three study groups according to previous treatment was done by Pearson chi-square test (or the maximum likelihood correction if small samples) for qualitative variables; for quantitative variables, ANOVA test was used if normal and the Kruskal-Wallis test otherwise.

Next, the differences in the different treatment groups, between the percentages at admission and at discharge, were analyzed using the McNemar Chi-square test, calculating in each case, the values ​​of the difference between the percentage at discharge minus the percentage at admission, and calculating the 95% confidence intervals (CI) of these percentage differences, if they had a value of 0, the binomial formula was used to calculate the 95% CI of the corresponding percentage. A two-tailed approach was used for all tests. Statistical significance was attained at *p* < 0.05.

All analyses were performed using SPSS v.28.0 software (IBM Corp. Released 2021. IBM SPSS Statistics for Windows, Version 28.0. Armonk, NY: IBM Corp) and y Epidat 4.2 (Epidat: program for epidemiological data analysis. V 4.2, 2016Ministry of Health, Xunta de Galicia, Spain; Pan American Health Organization (OPS-OMS); CES University, Colombia).

## Results

### Clinical characteristics of patients and groups

Some differences were found when comparing the clinical characteristics of the study subgroups in terms of lower age (*p* < 0.001) and lower proportion of patients with a history of some cardiovascular risk factors (hypertension, dyslipidemia, diabetes mellitus, renal failure, obesity) in the de novo HF subgroup without previous heart disease. In contrast, there was a higher percentage of smoking and active alcoholism in this group (*p* < 0.001).

The most frequently diagnosed underlying disease in all three groups was ischaemic heart disease. The second cause of disease was different according to the group. Thus, in the de novo HF group without previous heart disease, it was idiopathic dilated cardiomyopathy (24.1%), with previous heart disease atrial fibrillation/flutter (18.7%) and in the non-de novo group, valvular heart disease (18.9%) (Table 1).

**Table 1:** Clinical characteristics

	De novo without previous heart disease	De novo with previous heart disease	Non-de novo	*p*	Total
n	108	107	387		602
Age (years), mean ± SD	65.3 ± 16.0	72.0 ± 1.1	71.7 ± 12.1	< 0.001	70.6 ± 13.1
Sex (Female), n (%)	36(33.3)	31(29.0)	101(26.1)	0.321	168(27.9)
***Baseline heart disease***, *n* (%)*				< 0.001	
Ischaemic Valvular DCM AF/Flutter HT Other	32(29.6) 18(16.7) 26(24.1) 13(12.0) 4(3.7) 15(13.9)	48(44.9) 17(15.9) 13(12.1) 20(18.7) 4(3.7) 5(4.7)	175(45.2) 73(18.9) 67(17.3) 17(4.4) 20(5.2) 35(9.0)		255(42.4) 108(17.9) 106(17.6) 50(8.3) 28(4.7) 55(8.9)
***Antecedents***, *n (*%)					
Previous CVS HT Dyslipidaemia DM Active smoking Active drinking COPD SAHS Obesity Renal failure Hypothyroidism AF Stroke PVD	14(13.0) 41(38.0) 31(28.7) 28(25.9) 203(52.5) 13(12.0) 9(8.3) 3(3.3) 13(12.0) 20(18.5) 5(4.6) 39(36.1) 6(6.3) 4(4.5)	9(8.4) 91(85.0) 59(55.1) 54(50.5) 144(37.2) 8(7.5) 9(8.4) 7(7.4) 16(15.0) 85(79.4) 7(6.5) 37(34.6) 15(15.3) 8(8.5)	84(21.7) 314(81.1) 258(66.7) 196(50.6) 40(10.3) 17(4.4) 78(20.2) 66(21.1) 74(19.1) 209(54.0) 44(11.4) 222(57.4) 31(9.5) 38(12.1)	0.002 < 0.001 < 0.001 < 0.001 < 0.001 0.003 0.001 < 0.001 0.181 < 0.001 0.057 < 0.001 0.099 0.080	107(17.8) 446(74.1) 348(57.8) 278(46.2) 91(15.1) 38(6.3) 96(15.9) 76(15.3) 103(17.1) 220(36.5) 56(9.3) 298(49.5) 52(10.0) 50(10.1)

* Refers to the underlying etiology that prompts the diagnosis of heart failure, whether de novo or not

*Abbreviations*: AF: atrial fibrillation; CVS: cardiovascular surgery; COPD: chronic obstructive pulmonary disease; DCM: dilated cardiomyopathy; HT: hypertension; DM: diabetes mellitus; SAHS: sleep apnea-hypopnea syndrome; SD: standard deviation; PVD: peripheral vascular disease

When comparing the analytical values at admission in the three subgroups, no significant differences were found (Table 2).

**Table 2:** Analytical characteristics on admission

	De novo without previous heart disease	De novo with previous heart disease	Non-de novo	*p*	Total
Urea (mg/dL)*	56.5(37.0/84.8)	66.0(40.5/93.3)	55.0(39.0/91.0)	0.540	57(39/88.5)
Creatinine (mg/dL)#	1.49 ± 1.19	1.61 ± 0.95	1.62 ± 1.14	0.551	1.60 ± 1.12
GFR (ml/min/1.73m^2^)*	56(35/77.5)	45(32/71)	53(32/76)	0.224	52.5(33/75)
NT ProBNP (pg/mL)*	6238(2800/12,152)	8614(3541.5/18518.5)	7713.5(3460.5/15,470)	0.270	7464(3435/15,425)
Sodium (mEq/L)#	138.9 ± 5.2	138.9 ± 4.5	138.6 ± 4.4	0.778	138.7 ± 4.6
Potassium (mEq/L)#	4.32 ± 0.53	4.39 ± 0.70	4.39 ± 0.59	0.601	4.38 ± 0.60
Hemoglobin (g/dL)#	13.0 ± 3.2	13.5 ± 5.5	12.9 ± 3.1	0.284	13.0 ± 3.7
Hematocrit (%)#	39.3 ± 6.6	38.1 ± 8.2	38.8 ± 6.6	0.439	38.8 ± 6.9
Uric acid (mg/dL)#	8.1 ± 2.7	9.0 ± 6.0	8.3 ± 2.9	0.240	8.4 ± 3.6
Cholesterol-HDL (mg/dL)*	36(30/46)	36(28/45)	35(28/43)	0.412	36(29/44)
Cholesterol-LDL (mg/dL) *	75.5(58/99)	73(54/92)	71(54/95)	0.326	72(54/95)
Triglycerides (mg/dL)*	93(72.5/129.5)	97.5(74/124)	95(72/119.5)	0.670	95.5(73.0/120.3)
TSAT (%)*	17(13/26)	17(12/24)	18(13/24)	0.509	18(13/24)
Ferritin (ng/mL)*	193(109/378)	168(79/358)	163(89/335.5)	0.568	168(87/351)
HbA1c (%)#	6.3 ± 1.2	6.8 ± 5.7	6.3 ± 1.2	0.337	6.4 ± 2.6
CA125 (U/mL)*	90.4(36.9/226.3)	90.0(35.9/181.0)	89.9(47.2/200.5)	0.827	90.0(44.7/199.8)

* median (p25/p75)

# mean ± standard deviation

*Abbreviations*: CA125: carbohydrate antigen 125; GFR: glomerular filtration rate; HbA1c: glycated hemoglobin; NTproBNP: amino-terminal propeptide of B-type natriuretic peptide; TSAT: transferrin saturation

### Analysis by pharmacological groups

All pharmacological groups, and in all three years of the study, experienced an increase during admission, such that there were significant differences (*p* < 0.001) between the percentage of patients taking these drugs at admission and discharge. This was not the case for ACEIs/ARBs (*p* > 0.2) which reduced their prescription in favour of ARNI (*p* < 0.001).

In the global analysis (years 2020–2022) the pillar that most increased its prescription during admission was MRA (27.2%), followed by Bb (23.3%), SGLT2i (20.3%) and RAASI [includes ACEIs, ARB, and ARNI (17.3%)], all with *p* < 0.001. However, the pillar that increased the most in 2022 was SGLT2i (32.5%) followed by MRA (31.5) and Bb (25.2%). The least increase was RAASI (15.1%) as its largest increase occurred in 2020 (24.4%), with a reduction of ACEI and ARBs in favour of ARNI at discharge. The pharmacological group with the largest progressive increase over the study period was SGLT2i, from 20.0% (2020) to 70.4% (2022). These data, over the last 3 years as a whole and for each year, are shown in Table 3.

**Table 3:** Prescription by pharmacological groups

	2020 *n* = 135					
	ACEIARB	ARNI	RAASi	Bb	MRA	SGLT2i
Admission	41(30.4)	23(17.0)	63(46.7)	85(63.0)	46(34.1)	17(12.6)
Discharge	47(34.8)	50(37.0)	96(71.1)	103(76.3)	74(54.8)	27(20.0)
*p*	0.418	< 0.001	< 0.001	0.004	< 0.001	0.002
Difference % (CI95%)	4.4(-4.5/13.4)	20.0(11.8/28.2)	24.4(14.9/34.0)	13.3(4.9/21.7)	20.7(12.3/29.2)	7.4(1.4/8.1)
	2021 *n* = 261					
	ACEI/ARB	ARNI	RAASi	Bb	MRA	SGLT2i
Admission	98(37.5)	49(18.8)	147(56.3)	150(57.5)	99(37.9)	67(25.7)
Discharge	86(33.0)	101(38.7)	187(71.6)	220(84.3)	170(65.1)	112(42.9)
*p*	0.224	< 0.001	< 0.001	< 0.001	< 0.001	< 0.001
Difference % (CI95%)	-4.5(-11.4/2.2)	19.9(14.5/25.3)	15.3(9.2/21.4)	49.6(38.9/60.3)	27.2(21.2/33.2)	17.2(12.2/22.3)
	2022 *n* = 206					
	ACEI/ARB	ARNI	RAASi	Bb	MRA	SGLT2i
Admission	69(33.5)	40(19.4)	109(52.9)	103(50.0)	71(34.5)	78(37.9)
Discharge	60(29.1)	80(38.8)	140(68.0)	155(75.2)	136(66.0)	145(70.4)
*p*	0.253	< 0.001	< 0.001	< 0.001	< 0.001	< 0.001
Difference % (CI95%)	-4.4(-11.0/2.3)	19.4(13.1/25.7)	15.1(8.3/21.8)	25.2(18.6/31.9)	31.5(24.9/38.2)	32.5(26.0/39.1)
	2020–2022 *n* = 602					
	ACEI/ARB	ARNI	RAASi	Bb	MRA	SGLT2i
Admission	208(34.6)	112(18.6)	319(53.0)	338(56.1)	216(35.9)	162(26.9)
Discharge	193(32.1)	231(38.4)	423(70.3)	478(79.4)	380(63.1)	284(47.2)
*p*	0.281	< 0.001	< 0.001	< 0.001	< 0.001	< 0.001
Difference % (CI95%)	-2.5(-6.7/1.7)	19.8(16.1/23.4)	17.3(13.2/21.4)	23.3(19.1/27.4)	27.2(23.3/31.2)	20.3(16.9/23.6)

CI95%: Confidence Interval of 95%

*Abbreviations*: ACEI/ARB: angiotensin-converting enzyme inhibitors/angiotensin receptor blockers; ARNI: angiotensin receptor and neprilysin inhibitor; Bb: beta-blockers; MRA: mineralocorticoid receptor antagonists; RAASi: renin-angiotensin-aldoseterone system inhibitors. SGLT2i: sodium/glucose cotransporter type 2 inhibitors.

**Fig. 2:**
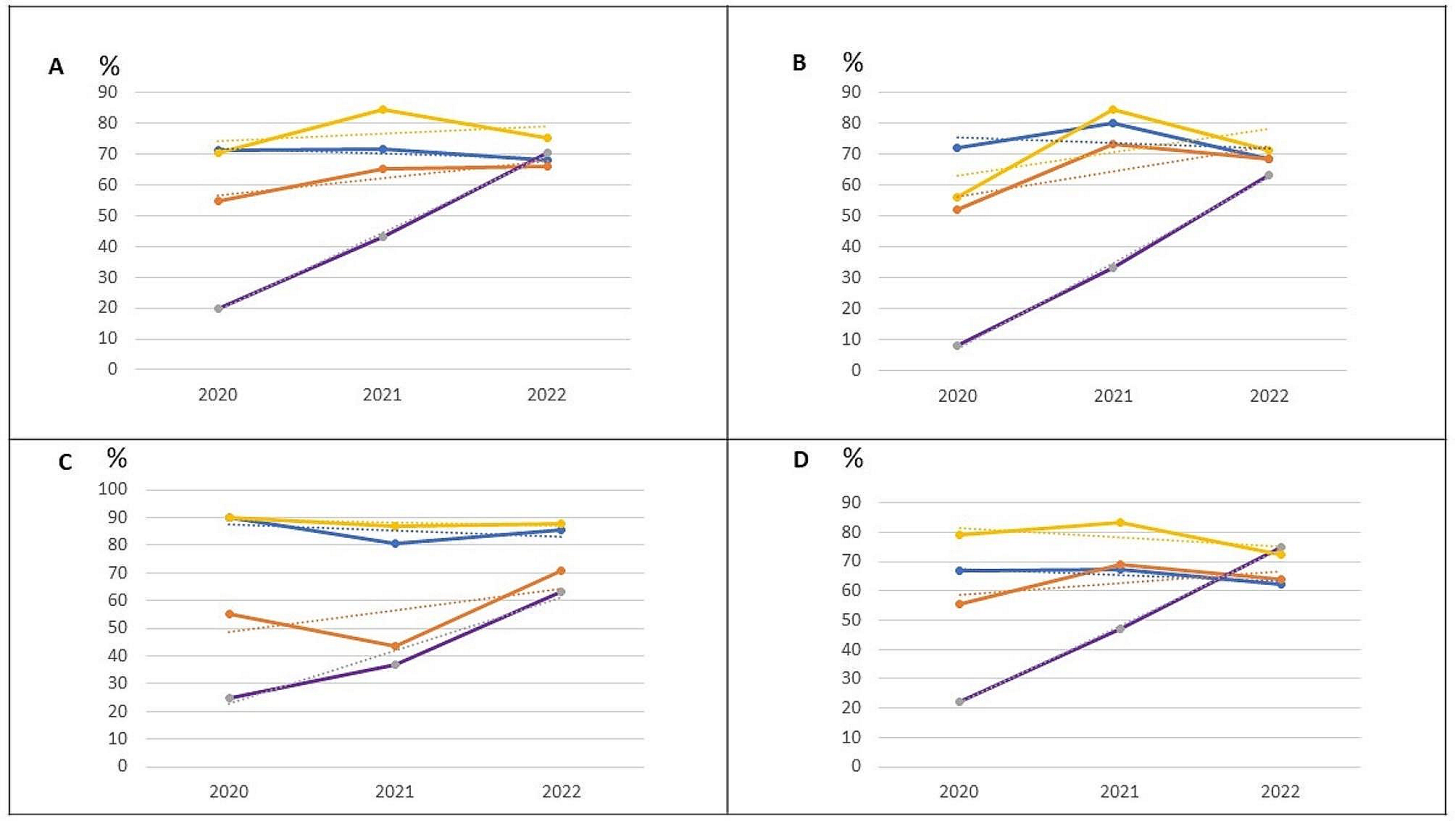
Evolution of pharmacological groups. (**A**) Global Series. (**B**) De Novo without previous heart disease. (**C**) De Novo with previous heart disease. (**D**) Non-de novo Percentage of patients with a prescription at discharge in the study drug groups. Blue line: RAASi, orange line: MRA, yellow line: Bb, purple line: SGLT2i. Dashed lines: linear trends

The trend lines show a rapid rise in the prescription rate at discharge of SGLT2i and a stabilisation of the RAASI pharmacological group (Fig. 2A).

### Study by heart failure subgroups

In the study period (2020–2022), patients with a diagnosis of de novo HF without previous heart disease who were admitted for decompensation were discharged mainly with RAASI (74.1%) and Bb (73.1%). The percentage of MRA administration was 66.7% and SGLT2i 38%. The largest progression in this group was the prescription of SGLT2i from 8% (2020) to 63.2% (2022). All differences were significant as they were not taking any of these drugs on admission. These data can be seen in Table 4; Fig. 2B.

**Table 4:** Prescription by pharmacological group and type of HF.

	2020																	
	De novo HF without previous heart disease						De novo with previous heart disease						Non-de novo					
	ACEI/ARB	ARNI	RAASI	Bb	MRA	SGLT2i	ACEI/ARB	ARNI	RAASI	Bb	MRA	SGLT2i	ACEI/ARB	ARNI	RAASI	Bb	MRA	SGLT2i
Admission	0	0	0	0	0	0	14(70.0)	0	14(70.0)	11(55.0)	3(15.0)	2(10.0)	27(30.0)	23(25.6)	49(54.4)	74(82.2)	43(47.8)	15(16.7)
Discharge	9(36.0)	9(36.0)	18(72.0)	14(56.0)	13(52.0)	2(8.0)	14(70.0)	4(20.0)	18(90.0)	18(90.0)	11(55.0)	5(25.0)	24(26.7)	37(41.1)	60(66.7)	71(78.9)	50(55.6)	20(22.2)
P	-	-	-	-	-	-	1	-	0.219	0.016	0.008	0.250	0.648	0.007	0.052	0.607	0.167	0.063
Difference % (CI95%)	36.0(15.2/56.8)	36.0(15.2/56.8)	72.0(52.4/91.6)	56.0(34.5/77.5)	52.0(30.4/73.6)	8.0(1.0/26.0)	0.0(-31.3/31.3)	20.0(5.7/43.7)	20.0(-8.5/29.7)	40.0(11.1/47.8)	40.0(7.8/48.8)	15.0(-17.8/27.2)	-3.3(-12.6/7.0)	15.5(5.4/25.7)	12.2(1.2/23.2)	-3.3(-11.2/5.9)	7.8(-2.8/15.8)	5.5(-1.5/8.5)
	2021																	
	ACEI/ARB	ARNI	RAASI	Bb	MRA	SGLT2i	ACEI/ARB	ARNI	RAASI	Bb	MRA	SGLT2i	ACEI/ARB	ARNI	RAASI	Bb	MRA	SGLT2i
Admission	0	0	0	0	0	0	35(76.1)	1(2.2)	36(78.3)	14(30.4)	1(2.2)	6(13.0)	63(37.1)	48(28.2)	111(65.3)	136(80.0)	98(57.6)	61(35.9)
Discharge	21(46.7)	15(33.3)	36(80.0)	38(84.4)	33(73.3)	15(33.3)	22(47.8)	15(32.6)	37(80.4)	40(87.0)	20(43.5)	17(37.0)	43(25.3)	71(41.8)	114(67.1)	142(83.5)	117(68.8)	80(47.1)
P	-	-	-	-	-	-	0.007	< 0.001	0.999	< 0.001	< 0.001	0.001	0.002	< 0.001	0.690	0.362	0.001	< 0.001
Difference % (CI95%)	46.7(31.0/62.4)	33.3(18.4/48.2)	80.0(67.2/92.8)	84.4(72.7/96.1)	73.3(59.3/87.3)	33.3(18.4/48.2)	-28.3(-46.0/-10.5)	30.4(11.5/31.8)	2.1(-12.7/15.9)	56.6(40.8/72.3)	41.3(25.9/56.8)	24.0(8.7/28.0)	-11.8(-18.8/-4.7)	13.5(7.2/19.8)	1.8(-4.0/7.5)	3.5(-2.8/9.8)	11.2(5.2/17.2)	11.2(5.4/16.9)
	2022																	
	ACEI/ARB	ARNI	RAASI	Bb	MRA	SGLT2i	ACEI/ARB	ARNI	RAASI	Bb	MRA	SGLT2i	ACEI/ARB	ARNI	RAASI	Bb	MRA	SGLT2i
Admission	0	0	0	0	0	0	30(73.2)	0	30(73.2)	15(36.6)	2(4.9)	9(22.0)	39(30.7)	40(31.5)	79(62.2)	88(69.3)	69(54.3)	69(54.3)
Discharge	12(31.6)	14(36.8)	26(68.4)	27(71.1)	26(68.4)	24(63.2)	17(41.5)	18(43.9)	35(85.4)	36(87.8)	29(70.7)	26(63.4)	31(24.4)	48(37.8)	79(62.2)	92(72.4)	81(63.8)	95(74.8)
P	-	-	-	-	-	-	0.004	-	0.227	< 0.001	< 0.001	< 0.001	0.096	0.115	1	0.424	0.004	< 0.001
Difference % (CI95%)	31.6(15.5/47.7)	36.8(20.2/53.5)	68.4(52.3/84.5)	71.1(55.3/86.8)	68.4(52.3/84.5)	63.2(46.5/79.8)	-31.7(-43.2/-9.7)	43.9(27.5/60.3)	12.2(-5.9/23.6)	51.2(39.2/63.1)	65.8(47.9/80.5)	41.4(25.6/57.2)	-6.3(-11.4/1.0)	6.3(-0.5/13.1)	0.0(-6.8/6.8)	3.1(-3.3/8.2)	9.5(2.9/12.2)	20.5(13.1/27.8)
	2020–2022																	
	ACEI/ARB	ARNI	RAASI	Bb	MRA	SGLT2i	ACEI/ARB	ARNI	RAASI	Bb	MRA	SGLT2i	ACEI/ARB	ARNI	RAASI	Bb	MRA	SGLT2i
Admission	0	0	0	0	0	0	79(73.8)	1(0.9)	80(74.8)	40(37.4)	6(5.6)	17(15.9)	129(33.3)	111(28.7)	239(61.8)	298(77.0)	210(54.3)	145(37.5)
Discharge	42(38.9)	38(35.2)	80(74.1)	79(73.1)	72(66.7)	41(38.0)	53(49.5)	37(34.6)	90(84.1)	94(87.9)	60(56.1)	48(44.9)	98(25.3)	156(40.3)	253(65.4)	305(78.8)	248(64.1)	195(50.4)
P	-	-	-	-	-	-	< 0.001	< 0.001	0.087	< 0.001	< 0.001	< 0.001	0.001	< 0.001	0.120	0.435	< 0.001	< 0.001
Difference % (CI95%)	38.9(29.2/48.5)	35.2(25.7–44.7)	74.1(65.3/82.8)	73.1(64.3/82.0)	66.7(57.3/76.0)	38.0(28.3/47.6)	-24.3(-36.4/-12.2)	33.7(24.8/42.7)	9.3(-0.2/18.9)	50.5(40.6/60.4)	50.5(40.6/60.3)	29.0(20.1/37.9)	-8.0(-12.4/-3.6)	11.6(7.3/15.9)	3.6(-0.6/7.8)	1.8(-2.1/5.7)	9.8(5.9/13.8)	12.9(9.2/16.6)

95% CI: 95% confidence interval.

*Abbreviations*: Bb: beta-blockers; IECA/ARB: angiotensin converting enzyme inhibitor / angiotensin receptor antagonist; ARNI: neprilysin and angiotensin receptor inhibitor; RAASI: renin angiotensin system inhibitor; MRA: mineralocorticoid receptor antagonist; SGLT2i: sodium-glucose cotransporter dual inhibitor

In the de novo group with previous heart disease, some drugs were already prescribed, before admission for decompensation, concerning their baseline disease. Mostly, ACEIs/ARBs (73.8%) and Bb (37.4%). During admission, all drugs showed a very significant increase in prescription (*p* < 0.001) except for ACEIs/ARBs, which decreased in favour of ARNI (*p* < 0.001). The largest progressive increase over the 3 years of study was with SGLT2i from 25% (2020) to 63.4% (2022) of prescribing at discharge. Table 4; Fig. 2C show the values obtained.

Patients already diagnosed with HF who were admitted also showed significant differences between drugs at admission and discharge. However, this was not the case in all groups as they had already been uptitrated according to previous guidelines. Even so, when analysing the study years (2020–2022) there was a significant reduction in ACEIs/ARBs in favour of ARNI (*p* < 0.001), an increase in the prescription of ARMs and SGLT2i (*p* < 0.001) with no change in the prescription of Bb (p:0.44). In this subgroup of patients, the drug that experienced the greatest progressive growth was SGLT2i, from 22.2% prescribing at discharge in 2020 to 74.8% in 2022. Data from the analysis of this subgroup can be seen in Table 4; Fig. 2D.

### Prescription of combined treatment. Overall trend and by subgroups

Globally, there was a significant increase in the prescription of the 4 pillars at discharge compared to admission (*p* < 0.001). This increase was progressive over the study years, from 7.4% at discharge (2020) to 32.5% (2022).

**Fig. 3:**
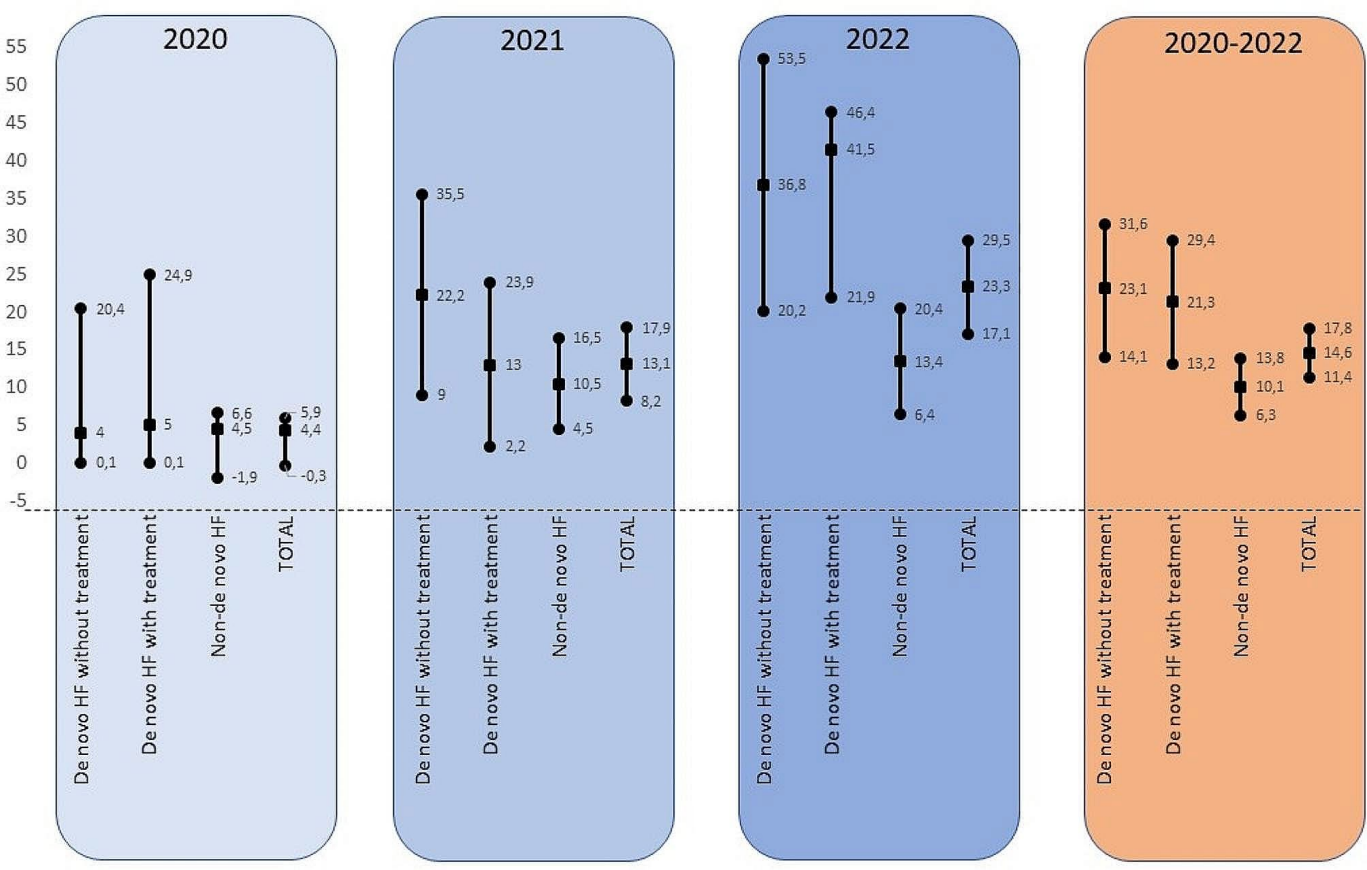
Evolution of the overall combined prescription and by subgroups Each line shows the percentage differences between admission and discharge, with their corresponding 95% CIs, by study group and by year Abbreviations: HF: heart failure

**Fig. 4:**
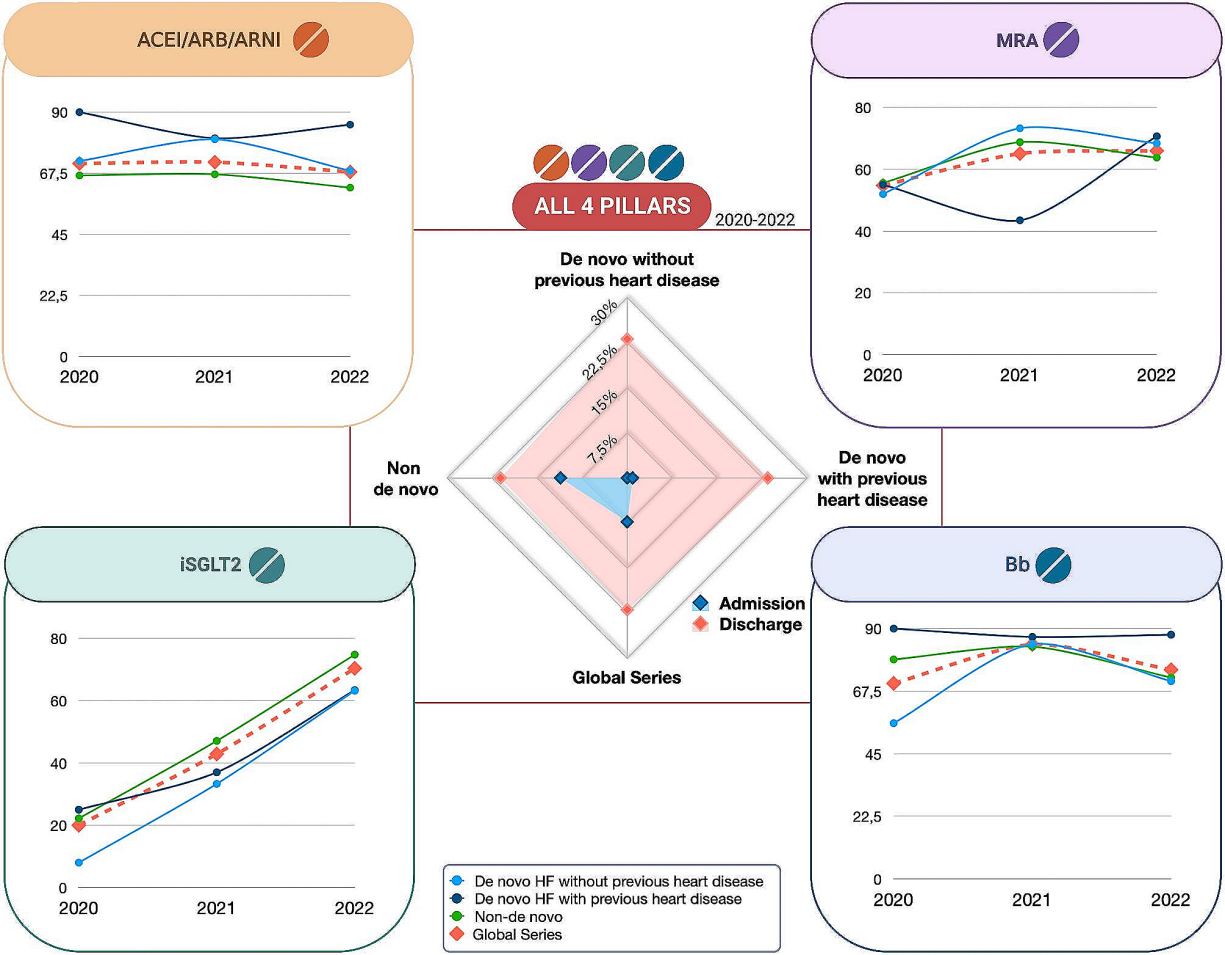
Summary of results Abbreviations: ACEI: angiotensin-converting enzyme inhibitors.; ARB: angiotensin receptor blockers; ARNI: neprilysin inhibitors; Bb: beta-blockers; HF: heart failure; MRA: mineralocorticoid receptor antagonists; SGLT2i: sodium/glucose cotransporter type 2 inhibitors

In all subgroups analysed, the annual progression was constant. However, the groups that increased the most over the entire series (2020–2022) were patients with de novo HF without previous heart disease (23.1%) and de novo HF with previous heart disease (22.5%). The group with the lowest increase in prescription at discharge was the non-novo group (10%) as they started from higher values. These data can be seen in Table 5; Fig. 3. An overall summary of these results can be seen in Fig. 4.

**Table 5:** Prescription of the combined treatment

	2020			
		De novo HF without previous heart disease	De novo with previous heart disease	Non-de novo
Admission	4(3.0)	0	0	4(4.4)
Discharge	10(7.4)	1 (4.0)	1(5.0)	8(8.9)
*P*	0.070	-	-	0.219
Difference % (CI95%)	4.4(-0.3/5.9)	4.0(0.1/20.4)	5.0(0.1/24.9)	4.5(-1.9/6.6)
	2021			
	Global	De novo HF without previous heart disease	De novo with previous heart disease	Non-de novo
Admission	21(8.0)	0	0	21(12.4)
Discharge	55(21.1)	10(22.2)	6(13.0)	39(22.9)
*P*	< 0.001	-	-	0.001
Difference % (CI95%)	13.1(8.2/17.9)	22.2(9.0/35.5)	13.0(2.2/23.9)	10.5(4.5/16.5)
	2022			
	Global	De novo HF without previous heart disease	De novo with previous heart disease	Non-de novo
Admission	19(9.2)	0	1(2.4)	18(14.2)
Discharge	67(32.5)	14(36.8)	18(43.9)	35(27.6)
*p*	< 0.001	-	< 0.001	0.001
Difference % (CI95%)	23.3(17.1/29.5)	36.8(20.2/53.5)	41.5(21.9/46.4)	13.4(6.4/20.4)
	2020–2022			
	Global	De novo HF without previous heart disease	De novo with previous heart disease	Non-de novo
Admission	44(7.3)	0	1(0.9)	43(11.1)
Discharge	132(21.9)	25(23.1)	25(23.4)	82(21.1)
*p*	< 0.001	-	< 0.001	< 0.001
Difference % (CI95%)	14.6(11.4/17.8)	23.1(14.1/31.6)	22.5(13.2/29.4)	10(6.3/13.8)

95% CI: 95% confidence interval.

*Abbreviations*: HF: Heart failure

## Discussion

Currently, scientific guidelines on HF advise administering the four pharmacological groups that have shown prognostic benefit (ACEI/ARB/ARNI, Bb, MRA, and SGLT2i) [1, 2] to patients with HFrEF [1, 2]. It is recommended that they are administered as soon as possible after diagnosis and at sufficient doses. Even the most recent drugs already have studies such as TRANSITION or DICTATE-AHF that prove their safety and efficacy immediately after an acute episode [7, 8]. The combined administration of these 4 pharmacological groups is usually completed after discharge. However, it would be advisable to try to prescribe them (even at low doses) before hospital discharge [9, 10]. This study aimed to analyze the evolution of prescription in recent years, the percentage of patients discharged from hospital with the 4 pharmacological groups, the evolution of each of them, and whether there are differences in prescription according to different HF subgroups. It was found that the pharmacological group whose prescription increased the most during the study period was SGLT2i, while the others remained stable. The HF group with the highest prescription of SGLT2i at discharge was non-de novo HF. Prescription at hospital discharge for all 4 pharmacological groups together increased over the study period to 32.5% in 2022. The subgroup with the highest combined prescription of the 4 pillars in 2022 was de novo HF with previous heart disease (43.9%).

In all HF subtypes, regardless of LVEF, hospitalisations are associated with worse short and long-term prognosis. Approximately one in four patients admitted for decompensation of HF dies or is readmitted within 30 days of discharge, with this prognosis becoming poorer as the number of readmissions increases [11, 12]. In turn, the time of admission is the most advisable time to educate the patient and an opportunity to incorporate all the recommended drugs with the possibility of performing dose escalation, evaluating in vivo tolerance to them, and the appearance of possible complications. All these results will provide us with a solid basis for adjusting and optimizing treatment as much as possible at discharge, without needing to wait for the first outpatient visit, and will allow a better transition from inpatient to outpatient care.

In most treatment optimization registries, patients who leave the hospital without achieving the maximum possible number of drugs take up to a year to incorporate the 4 pillars in the cases that manage to do so [13], with the prognostic detriment that this implies. It should be noted that, at present, these 4 pillars of treatment are mainly applied to HFrFE; to date, LVEF continues to be the fundamental parameter for the stratification of HF patients and their therapeutic management; the subgroup of HFrEF is the one with the largest therapeutic arsenal available with class I indication in clinical practice guidelines and therefore the one that generates the greatest controversy when deciding how and when to initiate the different drugs [1, 2, 14]. The percentage of patients who are discharged from the hospital with the 4 drugs is highly variable in the literature [5, 6, 15], since all patients admitted with decompensation of their HF are not the same. The implementation of the various pharmacological subgroups will be more difficult in patients with no previous prognosis-modifying treatment than in those who already have some pharmacological group as part of their usual treatment [16]. To avoid this possible bias, the groups selected to analyze the implementation of treatment at discharge in this study were patients with de novo HF without previous heart disease, de novo HF with previous heart disease, and non-de novo HF. The diagnosis of “*de novo* HF” was made when the patient was admitted for decompensation but had never been diagnosed with HF before, despite having cardiovascular risk factors or various heart diseases. The diagnosis of “acutely decompensated HF” was made when the patient had been diagnosed with HF on an outpatient basis, but had never been admitted for acute decompensation [17]. In this way, we stratified patients into treatment virgins and partially treated patients (as part of the treatment of their HF or as a treatment for another heart disease) in whom a smaller number of drugs had to be added to their usual therapy. The potential interactions and adverse effects inherent to drug use will be greater the more drugs added de novo, making it more difficult to maximise optimization in the de novo group without heart disease, as observed in the results obtained [14, 15].

Regarding the selected study period, it was taken into account that the last of the four pillars to demonstrate benefit was the SLGT2i group, whose first clinical trial (DAPA-HF) that demonstrated prognostic improvement in HF was published in November 2019 [4]. In this trial, the patients were already partially or totally treated with the other drugs with prognostic improvement and were not hospitalized patients. For this reason, the period selected in this study is limited to the last 3 years (2020–2022).

In terms of the clinical characteristics of the groups analyzed, differences were found in mean age, which was lower in the novo HF group without previous heart disease. In all three groups, the predominant underlying heart disease was ischemic, with nonischemic dilated cardiomyopathy being the second predominant etiology in the de novo group without previous heart disease, atrial fibrillation and flutter the second predominant etiology in the de novo group with previous heart disease, and valvular heart disease in the non-de novo group. These together represent the main etiologies seen in clinical practice in patients with HFrEF [18]. A high prevalence of multiple cardiovascular risk factors (CVRFs) was observed in all 3 groups, but with a tendency to present a lower percentage of CVRFs in the novo HF group without heart disease: a lower proportion of patients with a history of hypertension (HT), dyslipidemia, diabetes mellitus, and obesity. This profile is similar to that found in previous studies, in which classically patients with de novo HF have fewer CVRFs [17, 19]. Notably, renal failure and HT were much more prevalent in the non-de novo and de novo groups with previous heart disease.

In the analysis by pharmacological groups, we observed that all groups except for ACEI/ARB experienced a significant increase in their percentage at discharge concerning admission. This result was replicated during the three years. In the case of ACEI/ARB, this did not occur because they reduced their prescription in favor of ARNI, by the evidence available from the PARADIGM-HF study [1, 14]. It is for this reason that in the analysis by pharmacological groups we have separated ACEI/ARB from ARNI to analyze these two groups separately, and we added the RASSi group to see the total figure for this pharmacological group. This allows us to observe that, although in some subgroups there is a percentage drop in treatment with ACEI/ARB, overall the percentage increase in this pharmacological group (ARNI) is maintained. The increase at discharge that we observed in our results within all pharmacological groups, both annually and globally, is in line with the latest available evidence that shows that it is better to incorporate the maximum number of pillars possible in the treatment of HF than to prioritize increasing the doses of a smaller number of pharmacological groups [20].

If we analyze each pharmacological group individually, in the overall analysis of the years (2020–2022) the pillar that most increased its prescription in our study during admission was MRA, followed by Bb, SGLT2i, and RAASi. However, the pillar that increased the most in 2022 was SGLT2i, from 20.0% (2020) to 70.4% (2022). The trend lines show a rapid increase in the prescription of SGLT2i at discharge and a stabilization of the RAASi pharmacological group. Once again, it is clear that as new evidence emerges in favor of a pharmacological group such as SGLT2i (which have shown benefits not only in HF but also in other pathologies such as chronic kidney disease (CKD) [21, 22]) this evidence is transferred to real clinical practice, and it is important to know the real impact in our setting [23]. In 2022, a study conducted by our group aimed to compare, in a real-world setting, whether, in patients hospitalized for decompensated HF, the prescription of an SGLT2i during admission results in lower short-term morbidity and mortality. It was found that from 2019 to 2021, initiating treatment with SGLT2i in patients admitted to the hospital for acute heart failure was common [24]. These data are consistent with those found in the Spanish multicenter registry TIDY HF [6]. The TIDY HF analyzed the implementation of medical therapy in de novo heart failure with reduced ejection fraction and found that at 3 months of follow-up, 91.4% of patients were treated with SGLT2 inhibitors. Thus, at 3 months, almost 80% of patients reached quadruple therapy with a high implementation of SGLT2i. It should be noted that the only randomized clinical trial in this regard is the STRONG HF TRIAL, in which the SGLT2i pharmacological group was not included [25].

In the analysis according to the HF subgroup, it can be seen that in the de novo HF group without previous heart disease, patients were not taking any drugs at baseline, so the number of drugs to be incorporated to achieve the 4 pillars was greater. However, the prescription of the 4 pillars was not much lower than in the other groups: in 2022 more than 60% of these patients at discharge were receiving treatment with RAASi, MRA, Bb, and SGLT2i. Overall, these patients were the group whose values increased the most with respect to admission in the entire series (2020–2022). These results could be justified by the fact that some of the patients with previously diagnosed HF who, despite this, were not yet optimized to the maximum, had a contraindication, interaction, or poor tolerance to one of the pharmacological groups that make up the 4 pillars [26]. On the other hand, renal failure was less prevalent in the group of patients with de novo HF without previous heart disease, which undoubtedly facilitates the optimization of pharmacological treatment. Likewise, in this group, there was an exponential increase in the prescription of SGLT2i at discharge. In the group of patients with de novo HF with previous heart disease who were admitted, there were also significant differences between the drugs prescribed at admission and discharge. It can be observed that in this group very high percentages of prescriptions were reached at discharge, especially for RAASi and Bb, probably because most of these patients were already on treatment with these groups at admission. Even so, all drugs in this group showed a very significant increase in prescription, except for ACEI/ARB, which decreased in favor of ARNI. As in the previous group, and accordance with the available evidence [27, 28], the greatest progressive increase over the 3 years of the study occurred with SGLT2i, tripling of its prescription at discharge in 2022 compared to 2020.

The percentage of patients discharged from the hospital with all 4 drugs is highly variable in the literature and lower than desired in most studies [5, 29]. The results obtained in this analysis show an increasing trend year after year, even so, with lower than desired numbers of patients discharged with the complete combined treatment. Overall in the 3 HF groups, in 2020 only 7.4% of patients were discharged with all 4 drugs, being higher in the non-de novo group, in 2021 21.9%, being higher in the non-de novo group, and in 2022 32.5%, being higher in the de novo HF group with previous heart disease, indicating that even in these patients an early initiation of prognosis-modifying drugs is possible. Thus, although there is room for improvement, the percentage of patients on treatment with the 4 treatment pillars has been increasing, largely at the expense of the growth in the prescription of the SGLT2i pharmacological group. This fact is consistent with the increasing evidence of the prognostic benefit of these 4 pillars. Although the results obtained are far from the desired percentages, they are results of real clinical practice, which reflect the reality of prescribing in this complex pathology [5, 15, 16]. It would be of interest to analyze the impact of the different reasons why, in a certain percentage of patients, the 4 pharmacological groups are not implemented. Most likely, among these factors is hyperkalemia, that can impact the management of patients with HF by promoting the discontinuation of therapies, thus, negatively increasing the risk for mortality [30].

Among the limitations of the manuscript, it should be noted that it is a retrospective study. This type of design undoubtedly carries intrinsic limitations, such as the possibility of lacking some data, confounding variables, etc. On the one hand, it is a single-center study, which may limit external validity. On the other hand, the prescription was dependent on the physician responsible for the patient during admission, with possible variability of the professionals and the type of patient. It was not possible to assess whether the lack of prescription was due to any side effect or added comorbidity. Additionally, data on the educational level of the patients included in the study, as well as on the daily number of pills from the groups, has not been analyzed. These factors may have some influence on treatment adherence. However, this is countered by the fact that medical prescription has been analyzed, not specifically the adherence. Nevertheless, in contrast, this is a study of a large number of patients, from a single referral center in which the usual clinical practice is similar to that of all the clinical cardiologists attending these patients. In addition, the clinical trials show very high prescription figures but these are not transferable, due to the design of the studies, to the real world where the percentage of prescription of these patients is rather low. Finally, it should be noted that the data were systematically entered into the database at patient discharge by the same cardiologists who are experts in the management of these patients (cardiologists belonging to the HF Unit), which minimizes errors in data collection.

## Conclusions

In conclusion, the percentage of patients with HFrEF admitted for acute HF and discharged with the combination of all drugs with prognostic improvement has progressed greatly in recent years, although in 2022 it was still low. The subgroup of de novo HF with previous heart disease showed the greatest optimization. The pharmacological group with the greatest increase in prescription over the 3-year study period was SGLT2i. Although in all pharmacological groups there were relevant differences between prescription at admission and discharge, prescription during admission should be further optimized to achieve greater prognostic improvement in patients.

## Data Availability

The datasets used and/or analysed during the current study are available from the corresponding author on reasonable request.

## Abbreviations

ACEI: Angiotensin-converting enzyme inhibitors
ARB: Angiotensin receptor blockers
ARNI: Neprilysin inhibitors
Bb: Beta-blockers
CVRF: Cardiovascular risk factors
HF: Heart failure
HFrEF: Heart failure with reduced ejection fraction.HT:Hypertension
LVEF: Left ventricular ejection fraction
MRA: Mineralocorticoid receptor antagonists
RAASi: Renin-angiotensin-aldosterone system inhibitors
SGLT2i: Sodium/glucose cotransporter type 2 inhibitors
